# Refining the Definition of US Safety-Net Hospitals to Improve Population Health

**DOI:** 10.1001/jamanetworkopen.2019.8562

**Published:** 2019-08-02

**Authors:** Tyler N. A. Winkelman, Katherine Diaz Vickery

**Affiliations:** Division of General Internal Medicine, Department of Medicine, Hennepin Healthcare, Minneapolis, Minnesota; Hennepin Healthcare Research Institute, Minneapolis, Minnesota.

## Abstract

Safety-net hospitals (SNHs) in the United States provide care for individuals and families regardless of their ability to pay.^1^ Since 1986, SNHs have received supplemental federal compensation through Medicare Disproportionate Share Hospital (DSH) payments. These payments have historically been calculated based on the proportion of hospital days accounted for by Medicare Supplemental Security Income plus Medicaid, non-Medicare inpatient days. The Affordable Care Act (ACA) modified this definition and reduced DSH payments to offset a growing insured, low-income population.^2^

## SNH Definitions Matter

In *JAMA Network Open*, Popescu et al^3^ highlight the implications of modifying the definition of SNHs used by the Centers for Medicare & Medicaid Services to allocate DSH payments. The authors examined concordance among SNH definitions based on the traditional Medicare DSH index and 2 commonly used contrasting definitions of safety-net status, the proportion of inpatient stays that were uninsured or paid by Medicaid and the cost of uncompensated care. They defined SNHs as those in the top quartile of each definition and found that each definition isolated a unique group of hospitals with limited overlap. Their results demonstrate that the definition of SNH is not merely a semantic policy detail; rather, it defines the purpose of the program and could determine the financial viability of hundreds of US hospitals.

Hospitals classified as SNHs under the traditional DSH formula were more likely to be larger, urban teaching hospitals. In contrast, SNH hospitals defined by Medicaid and uninsured caseload or uncompensated care were smaller and more rural and offered fewer services. Hospitals under the latter definitions were less financially stable, had larger unreimbursed costs from public payers, and incurred larger amounts of bad debt compared with SNHs under the traditional DSH formula.

## Align DSH Payments With National Health Priorities

The ACA-mandated changes to the DSH formula required uncompensated care be factored into payments, which became effective in 2018. Because smaller, rural hospitals are more likely to incur uncompensated costs, many of these hospitals will likely receive increased payments. This increase may slow the high rate of rural hospital closures in recent years.^4^ On the other hand, larger urban teaching hospitals may experience declines in DSH payments. Given these competing priorities, measuring the value provided to the US population by new DSH-recipient hospitals will be important.

We believe the success of the DSH program should be judged by its ability to make gains toward national health priorities. Ideally, these priorities stem from an existing framework like the forthcoming Healthy People 2030, which sets goals and objectives to improve health and reduce health inequities within the United States.^5^

For example, one proposed Healthy People 2030 objective aims to “reduce the proportion of persons who are unable to obtain or delay in obtaining necessary medical care.”^5(p15)^ Current changes to incorporate uncompensated care into the DSH formula may align with this goal, because they would increase funding to smaller rural hospitals that improve access for rural populations. Alternatively, Popescu and colleagues^3^ show that a definition that combines Medicaid and uninsured inpatient days would disproportionately favor hospitals with maternity care. This alternative definition would support Healthy People 2030 proposed objectives to “reduce severe maternal complications of pregnancy identified during labor and delivery hospitalizations.”^5(p42)^

## Evidence-Based Policy

If the goal of DSH payments is to meet our national priorities, then the formula must be revisited on a regular basis, perhaps at 10-year intervals to correspond with the Healthy People initiative. This approach could provide time for payment strategies to take effect but create a process to ensure the policy remains nimble to emerging priorities. Such an approach is drawn from recent calls to develop processes to deprescribe policies that do not meet their stated goals.^6^ Rigorous, systems-based evaluation would be required to inform updates to the definition, a function for which the Agency for Healthcare Research and Quality is well suited.

As Popescu et al^3^ highlight, underreimbursement, the gap between what a public insurer (eg, Medicaid) pays and the costs incurred by a hospital to provide care, remains a key omission in the DSH formula. Under the current definition, hospitals that primarily provide care for patients with Medicaid could have large deficits due to underreimbursement but receive small DSH payments if they provide little uncompensated care. This problem has been documented in California, where lower Medicaid DSH payments after the ACA’s Medicaid expansion are expected to leave a gap of more than $1 billion in funding for SNHs.^7^ Hospitals that care for many people with Medicaid may continue to face financial challenges without changes to Medicaid payments or additional changes to the DSH formula.

## Future Directions

Although the present study provides helpful evidence for policy makers implementing DSH reforms, a number of important areas for research remain. First, we must determine the degree to which DSH payments help hospitals expand clinical services aligned with the needs of the population. Second, we must examine whether DSH payment methods are associated with changes in the high rate of rural hospital closure in the United States. Third, we must estimate the effect of DSH payments on SNH investments to address social determinants of health.

Finally, DSH payments to reimburse uncompensated care would be largely unnecessary if health insurance coverage in the United States did not lag behind that of other industrialized countries. Despite historic gains in health insurance coverage since passage of the ACA, approximately 30 million US individuals were uninsured in 2018. High rates of uninsurance, relative to international standards, continue to create significant financial pressure for hospitals that strive to provide high-quality care to individuals and families regardless of their ability to pay. Without stable and comprehensive insurance for the US population, many SNHs will likely continue to rely on DSH funding to remain financially viable.

## Conclusions

Modifying the definition of SNH may dramatically change which hospitals receive supplemental DSH payments. Whether an evolving SNH definition can advance national health priorities remains to be seen. Independent evaluation of this policy is critical to ensure that hospitals, whose missions align with critical public health goals, remain financially viable.
